# Species–Habitat Associations in a Sri Lankan Dipterocarp Forest

**DOI:** 10.1111/jvs.70049

**Published:** 2025-07-17

**Authors:** G. J. M. Shanika R. Jayasinghe, Pushpakanthie Wijekoon, Tithira Lakkana, C. V. Savitri Gunatilleke, Sisira Ediriweera, Thorsten Wiegand

**Affiliations:** 1 Faculty of Applied Sciences, Uva Wellassa University, Badulla, Sri Lanka; 2 Department of Statistics and Computer Science, Faculty of Science, University of Peradeniya, Peradeniya, Sri Lanka; 3 Department of Botany, University of Peradeniya, Peradeniya, Sri Lanka; 4 Department of Ecological Modelling, Helmholtz Centre for Environmental Research-UFZ, Leipzig, Germany

**Keywords:** environmental heterogeneity, habitat preference, mixed-dipterocarp, soil nutrients, spatial point process theory, topography, species diversity, tree species

## Abstract

**Questions:**

A non-random spatial distribution of species in relation to environmental factors is an important mechanism for maintaining high tree diversity in tropical forests. Niche theory predicts that competing species should use the environment differently. However, we have only limited information on the extent to which environmental dependence and the relative importance of different types of environmental factors vary between species. It is also unclear how the environmental dependence differs according to life stage and species.

**Location:**

Here we investigate the environmental variables that determine the spatial distribution of tree species in a 25-ha plot of mixed-dipterocarp tropical rainforest in Sri Lanka.

**Methods:**

We compiled data on the spatial distribution of recruits, saplings, and adults of 57 tree species, as well as topographic and soil variables, and applied methods of spatial point process theory to estimate parametric spatial intensity functions for each life stage of the species as a function of environmental variables.

**Results:**

Most species distributions were significantly associated with at least one environmental variable, with elevation and the first principal component of soil nutrients being the most important ones. With few exceptions, species showed an intermediate strength of environmental dependence, and we observed a striking similarity in the intraspecific environmental dependence between life stages. Finally, only a few species showed for the same life stage strong positive or negative correlations in their intensity functions, while most species pairs showed only weak or no correlations at all.

**Conclusions:**

Taking together, our results indicate that the distribution of most of the more abundant species in our forest plot is influenced by local heterogeneity in environmental conditions, and that their environmental preferences lead to a spatial arrangement where competing species use the environment somewhat differently. Overall, our study provides a nuanced understanding of the complex environmental dependencies that shape tropical rainforest ecosystems at local spatial scales.

## 1 Introduction

Tropical rainforests are characterized by an exceptionally high diversity, and various mechanisms have been proposed to explain the maintenance of their high richness of woody species (Wright 2002). One of these mechanisms is niche differentiation, which is based on the relationship between species and environmental conditions, with species specializing along gradients of resource availability to avoid competitive exclusion (Wright 2002; Silvertown 2004). Ample evidence suggests that the smaller-scale distribution patterns of species (e.g., at scales of tens of ha) are influenced, among other factors, by local abiotic conditions such as topographic and edaphic factors (Harms et al. 2001; Gunatilleke et al. 2006; Comita et al. 2007; Kanagaraj et al. 2011; Allié et al. 2015; Guo et al. 2017; Liu et al. 2018). Topography is a first-order control of the smaller-scale spatial distribution of soil moisture (Sørensen et al. 2006; Kanagaraj et al. 2011), and it drives the amount of direct and diffuse radiation that codetermines the available light (Larsen and Speckman 2004). Additionally, several studies (e.g., Paoli et al. 2006; John et al. 2007; Baldeck et al. 2013; Allié et al. 2015) indicate that the distribution of species is related to soil nutrients. For example, John et al. (2007) found that the spatial distributions of a substantial proportion of neotropical tree species in three forest plots showed strong associations with soil nutrient distributions, and Baldeck et al. (2013) reported that both soil resources and topography accounted for significant and approximately equal variation in local tree community composition in eight large tropical forest plots.

The association of species with local abiotic conditions may change with their life stage (Webb and Peart 2000; Comita and Engelbrecht 2009; Lai et al. 2009; Kanagaraj et al. 2011; Shen et al. 2013; Liu et al. 2018). Following the idea of the regeneration niche (Grubb 1977), the observed associations of species distributions with edaphic factors are expected to arise during early life stages (Comita et al. 2007). If subsequent survival is primarily determined by environmental conditions (Comita and Engelbrecht 2009), species–habitat associations should strengthen with increasing individual size, particularly when seeds are dispersed away from the optimal habitat occupied by adult trees (Webb and Peart 2000). In contrast, if seeds are dispersed into the same habitat as their parents (e.g., due to localized dispersal), the spatial distributions of offspring and adults are expected to exhibit similar associations with environmental conditions (Zhu et al. 2013). However, if survival in later life stages is dominated by negative density-dependent mechanisms or by stochastic disturbances, the spatial distribution of species should be less and less determined by environmental conditions as the size of the individuals increases (Schupp 1992; Condit et al. 1994; Webb and Peart 2000; Kanagaraj et al. 2011). Finally, ontogenetic shifts in resource requirements, such as light (Lusk 2004; Poorter et al. 2005) or shifts in water use (Donovan and Ehleringer 1992), may cause additional changes in habitat requirements with size.

Many previous studies of species–habitat associations in forests were based on a priori fixed discrete habitats (e.g., Plotkin et al. 2000; Webb and Peart 2000; Harms et al. 2001; Itoh et al. 2010; Gunatilleke et al. 2006; Comita et al. 2007; Lai et al. 2009; Guo et al. 2017; Liu et al. 2018), which were often developed from continuous habitat variables, such as elevation, slope, or soil nutrient maps. However, it is sometimes difficult to define appropriate breakpoints for continuous habitat variables (Itoh et al. 2010), and species may respond in their performance to smooth gradients in environmental conditions rather than to abrupt changes in habitat type. An alternative is to use well-established methods from point process theory to estimate the intensity function *λ*(*x*), which describes the expected local density of individuals within a small area around the location *x* in the plot in a parametric way based on log-linear regression models (Wang et al. 2011; Baddeley et al. 2015) depending on continuous environmental variables, such as elevation, slope, or soil nutrients. Note that this approach differs from non-parametric intensity estimation based on kernel methods. Our parametric approach allows the identification of the contribution of the different environmental variables to the intensity function, and statistical tests such as the Berman test (Berman 1986; Wiegand and Moloney 2014) allow us to quantify the overall strength of association of the species distribution pattern to the estimated intensity function. The approach of estimating the intensity function is closely related to predictive habitat distribution models (Elith and Leathwick 2009) and allows the mapping of the fitted intensity function that describes the local habitat suitability for the species.

The general objectives of this study are to identify environmental variables that determine the smaller-scale distribution (i.e., the intensity) of different tree species in a mixed-dipterocarp tropical rainforest in Sri Lanka, and to assess whether the influence of environmental factors varies for the same species with life stage and between species in the same life stage. More specifically, we ask (i) which and how many environmental variables influence the intensity function of the different species significantly, (ii) how the proportion of specialist versus generalist species varies among species, (iii) if the environmental dependency of individual species changes with life stage, and (iv) to which degree the environmental dependencies of pairs of species in the same life stage are correlated.

Based on previous work in the lowland rainforests, Sri Lanka (e.g., Gunatilleke et al. 2006; Punchi-Manage et al. 2013, 2014) and its strong environmental heterogeneity, we expect that the local species distribution of most species will be influenced by topographic variables. Additionally, based on studies in other tropical forests (e.g., Paoli et al. 2006; John et al. 2007; Baldeck et al. 2013; Allié et al. 2015), the distribution of species should be related to soil nutrients (Question i). Nevertheless, we expect that other spatially structured mechanisms, such as seed dispersal, will partly counteract the species associations to environmental variables (Punchi-Manage et al. 2013), which should lead mostly to species with a moderate strength of environmental dependency and few generalist and specialist species (Question ii). As suggested by work in other tropical forests (e.g., Webb and Peart 2000; Comita and Engelbrecht 2009; Lai et al. 2009; Kanagaraj et al. 2011; Shen et al. 2013; Liu et al. 2018), we expect that environmental dependence should change with life stage, but environmental heterogeneity and dispersal limitation may lead only to moderate changes among life stages (Zhu et al. 2013; Question iii). Finally, niche theory lets us expect that all species should use the environmental heterogeneity of the plot somewhat differently (i.e., mostly moderate correlation between their intensity functions; Question iv).

## 2 Methods

### 2.1 Study Site

**Figure 1:**
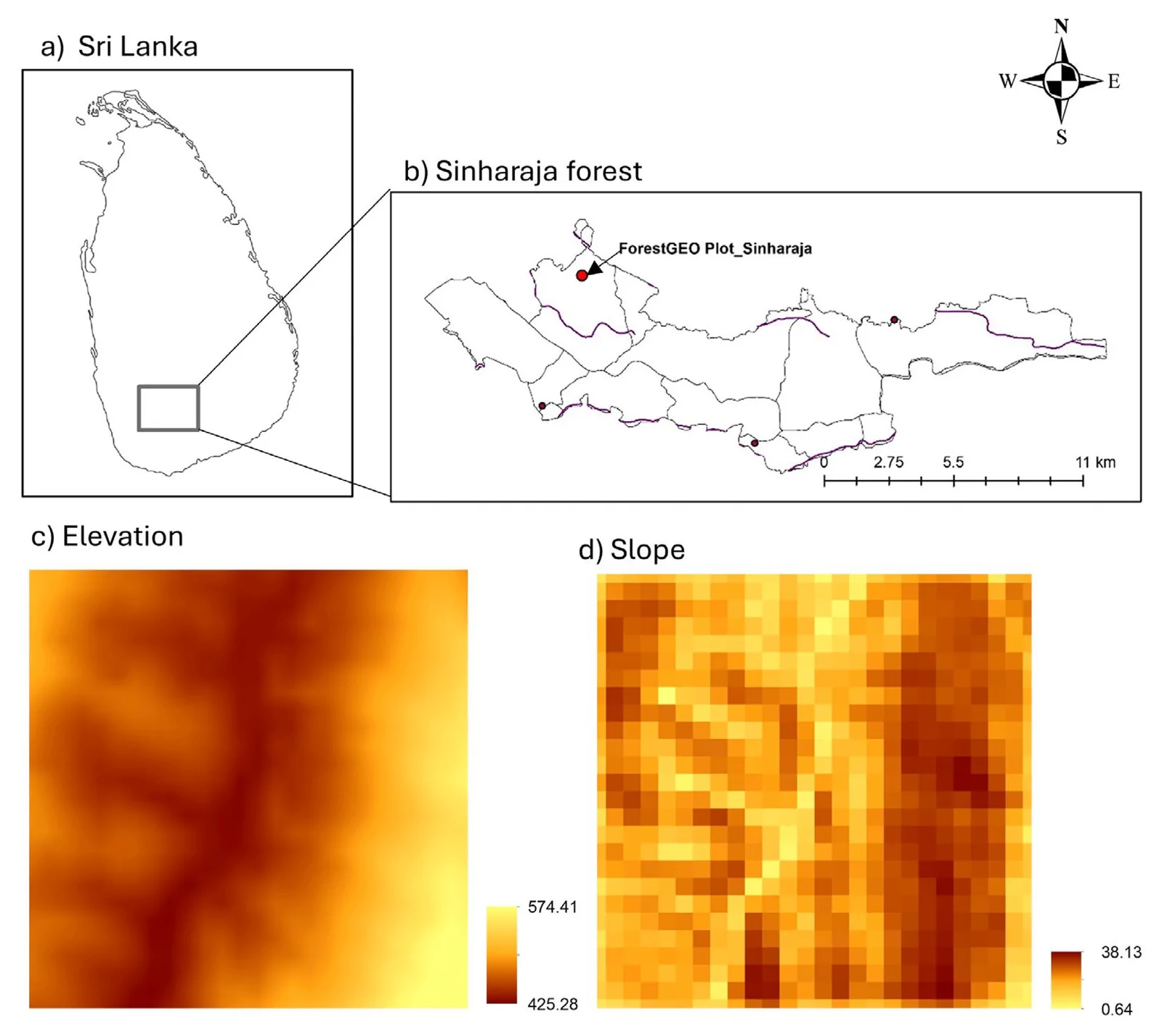
Location of the 25-ha Sinharaja FDP (a), the Sinharaja UNESCO World Heritage Site (b), an elevation map of the 25-ha plot (c), and a map of the slope (d).

The study was conducted in the 25-ha (500 × 500 m) Sinharaja forest dynamics plot (6° 21–26′ N, 80° 21–34′ E) (Figure 1), which is part of the ForestGEO network (Davies et al. 2021). The plot is representative of the ridge–steep slope–valley landscape of the lowland and mid-elevational rain forest, southwest Sri Lanka. The Sinharaja forest dynamics plot (Sinharaja FDP) is among the most topographically heterogeneous plots in the ForestGEO network (Gunatilleke et al. 2006), with elevation ranging from 424 to 575 m above sea level (Figure 1c). The plot shows a central valley, which is bounded by a steeper slope facing southwest, reaching up to 575 m and a less steep slope facing northwest, rising to 505 m.

The Sinharaja FDP was established in 1993 and divided into 625 quadrats of 20 × 20 m (400 m^2^) each. The plot was censused five times, and all freestanding stems with a diameter at breast height (dbh) larger than 1 cm were measured, and each stem was mapped and identified to species. The dispersal mechanisms of the studied tree species are listed in Appendix S1. Three quarters of the species analyzed here are dispersed by animals; however, several of the dominant species, such as *Messua nagassarium* and *Shorea affinis* show dispersal limitation where seeds fall near parent trees (Appendix S1).

### 2.2 Vegetation Sampling and Size Classes

Our analysis incorporated all living trees, but excluding shrub species. First, we classified, for each census separately, the living trees of each species into the classes of adults, juveniles, and recruits. Individuals that appeared for the first time in a census (except the first census) were categorized as recruits, as they had crossed the size threshold of dhh = 1 cm. Next, we defined non-recruits as adults if their diameter at breast height (dbh) fulfilled the condition dbh ≥ dbh_99_^2/3^ (Bagchi et al. 2011), and as juveniles if dbh < dbh _99_^2/3^, where dbh_99_ is the 99^th^ percentile of the dbh distribution of the species. To increase the sample size, we merged for each life stage the individuals of different censuses, but we considered individuals that appeared in multiple censuses in the same life stage only once. For further analysis, we used species that comprised at least 50 recruits, 50 juveniles, and 50 adults. Based on these requirements, a total of 57 species were used in the study (Appendix S1). For most of the species, the highest number of individuals occurred in the juvenile stage and the lowest number in the recruit stage (Appendix S1). In total, our analysis used data from 197,817 trees.

### 2.3 Environmental Variables

To evaluate the effect of the environment, 6 topographic variables and 17 soil variables were used (Appendix S2). Soil analysis was conducted at the Smithsonian Tropical Research Institute Laboratory, Panama. The topographic variables included elevation, slope, aspect, curvature, topographic wetness index (TWI) (Sørensen et al. 2006), and altitude above the channel network (ACH). The topographic variables were estimated with SAGA GIS (http://www.saga-gis.org; for details see Appendix S3). We obtained 17 soil variables for all 20 × 20 m quadrats by applying the ordinary kriging method (John et al. 2007) (Appendix S2). Pairwise correlations between the topographic variables were high (Appendix S4). We therefore reduced the high dimensionality of the soil variables by conducting a principal component analysis and using the first components as variables that accounted for more than 75% of the variance. The calculation was done using the “krig()” function attached to the “fgeo.tool” package (https://github.com/forestgeo/fgeo.tool) in R Studio 1.2.5033.

### 2.4 Statistical Analysis

The focus of our analysis is the intensity function *λ*_*f*_(*x*) of a given life stage of a given focal species *f*, which characterizes the density of trees around a given location *x*, as driven by environmental heterogeneity. The quantity *λ*_*f*_(*x*) *dx* is the expected number of trees of the given life stage of the focal species *f* in a small region of area *dx* around location *x*. We approximate the intensity function based on 20 × 20 m quadrats and consequently the sum of *λ*_*f*_(*x*) *dx* over all 625 quadrats of our plot equals the total number *N*_*f*_ of focal individuals. The average density of focal individuals is given by *λ*_*f*_ = *N*_*f*_/*A* where *A* is the area of the plot (=25 ha).

Point process theory allows us to estimate the intensity functions non-parametrically or parametrically. The non-parametric approach is based on kernel methods, and the estimated intensity function is basically a smoothed version of the individual locations of the species (Baddeley et al. 2015). However, we were here interested in the influence of environmental variables on the spatial distribution of the tree individuals (Question i). We therefore used a parametric method to estimate the inensity function *λ*_*f*_(*x*) of species *f* at location *x*. To this end, we used a well-established multiple log-linear regression model (Equation 1), which fits the intensity function of each life stage of each focal species based on the observed tree locations and a number of environmental variables hypothesized to influence the distribution of the species (Wang et al. 2011; Baddeley et al. 2015). To represent the heterogeneity of the environment, we used topographic variables and principal components of soil nutrients as independent variables:

(1) $$\lambda_{f}(x)=\text{exp}\left(c_{f,0}+c_{f,1}x_{1}(x)+\ldots +c_{f,k}x_{k}(x)\right)$$

where *x*_*k*_(*x*) is the value of the *k*th environmental variable at location *x*, and the *c*_*f*_,_*k*_ are regression coefficients to be estimated. The estimated intensity function *λ*_*f*_(*x*) is a measure of local habitat suitability for species *f*, as it predicts the number of individuals in the unit area around location *x* based on our environmental variables. We refer to a species that tends to have a homogeneous intensity function (i.e., the values of *λ*_*f*_(*x*) are all very similar to the average density *λ*_*f*_ in the plot) as a generalist species because it shows little environmental dependence, and we refer to a species in which the intensity function varies greatly in space (i.e., it shows many quadrats of low suitability and some quadrats of high suitability) as a specialist species.

To select the most parsimonious intensity model for each species’ life stage, we first estimated Pearson’s correlation coefficient between all pairs of environmental variables (topographic variables and the three principal components of the soil nutrients variables), and then removed variables that were highly correlated (*r* > 0.5) to others and produced a Variance Inflation Factor (VIF) greater than five. Finally, we performed a stepwise model reduction of our log-linear model using Wald tests to retain only significant variables (Wang et al. 2011). Data analysis was conducted in R Studio 1.2.5033.

To respond to our questions, we conducted several analyses of the resulting intensity functions *λ*_*f*_(*x*). In a first analysis of the intensity function, we used the Berman test (Berman 1986; Wiegand and Moloney 2014) to determine the significance and the strength of the association of the observed species pattern to the intensity function predicted by the environmental variables (Question i). This analysis allows us to investigate the effect of other mechanisms, such as dispersal limitation, which may cause locally higher densities of the focal species that are unrelated to the environmental heterogeneity. Our method is based on the toroidal shift null model that tests whether a species pattern is independent of the intensity function, while conserving the observed spatial autocorrelation (aggregation) of the species locations in good approximation (Wiegand and Moloney 2014). We assessed significant deviations of independence at *α* = 0.05. The Berman test was conducted with the software Programita (http://programita.org; Wiegand and Moloney 2014). For details, see Appendix S5.

Second, to allocate a given species within the specialist–generalist gradient (Question ii), we estimated the proportion of individuals for which the intensity function predicts that they will be placed in quadrats with intensities below 1.5 times the average intensity. This threshold was suitable for the specialist–generalist assessment (see Appendices S6–S9). A generalist species shows little variability in habitat suitability across the plot (i.e., similar values of the intensity function) and therefore most individuals are predicted to be located in quadrats with intensities below this threshold. Conversely, a high proportion of individuals of a strong specialist species is predicted to be located in high-intensity quadrats (i.e., above the 1.5 threshold).

Third, to quantify the similarity in the predicted environmental dependence of individual species across life stages (Question iii) and among different species in the same life stages (Question iv), we calculated the correlation coefficients between the intensity function of pairs of species for each life stage, and for conspecifics between life stages. In the case of a high positive correlation, the two species or life stages show very similar environmental dependence, whereas a strong negative correlation points to a contrasting environmental dependence.

## 3 Results

### 3.1 Selection of Environmental Variables

The majority of the 17 soil variables were significantly and strongly correlated (> 0.7) (Appendix S4). The first three components of the PCA had eigenvalues greater than one and together accounted for 77.8% of the variance of all soil variables. They were extracted and used as variables representing the soil variables in our set of environmental variables (Appendices S10d–f and S11). The loadings of the 17 soil variables for the first 3 principal components indicated a clear separation into three groups (Appendix S12), where a first group was mostly associated with negative loadings of pH and base saturation, a second group with high positive loadings of Al, Fe, K, Rb-NO_3_, and Na, and a third showed high positive loading of Ca, Mn, and Mg. The majority of the Pearson correlation coefficients between environmental variables (i.e., topographic variables and principal components of soil) were small (< 0.5), but ACH was correlated with several other topographic variables, specifically TWI (−0.5) and Curvature (0.57) (Appendix S4), which caused VIF > 5. When removing ACH, all best-fitted intensity models showed VIF < 5. Taking all together, we used for the intensity modeling five topographic variables (elevation, slope, aspect, curvature, and TWI) and the first three soil PCs (Appendix S10).

### 3.2 Influence of Environmental Variables on the Intensity Function (Questions i and iii)

**Figure 2:**
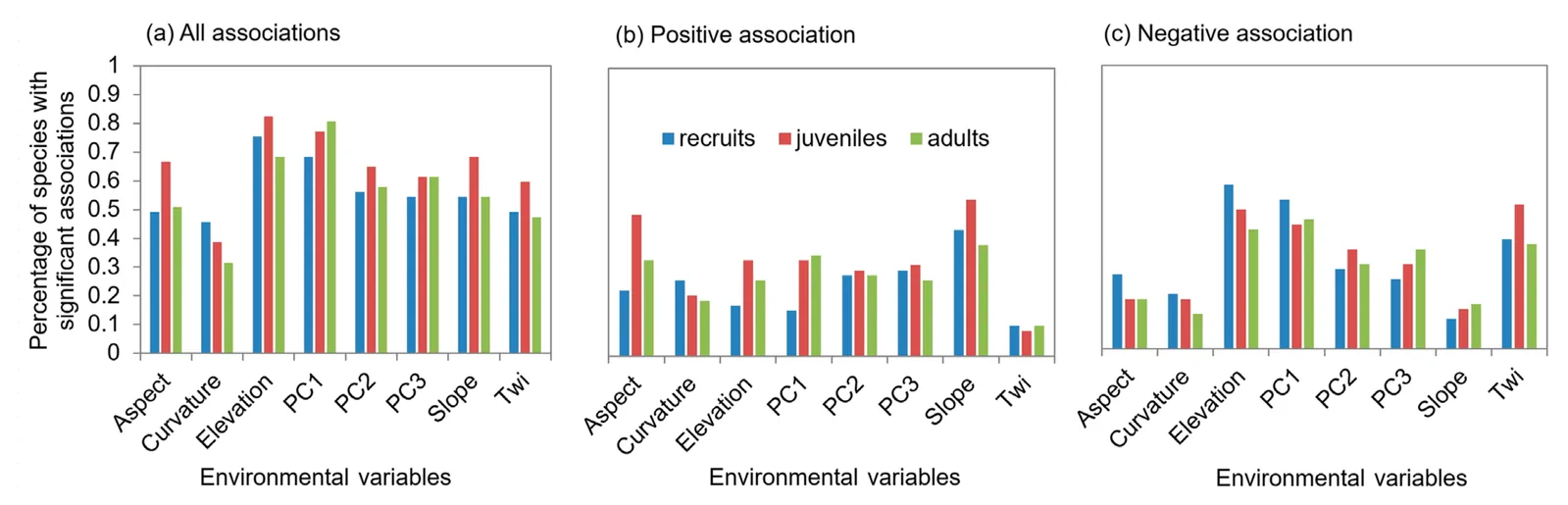
Influence of the different environmental variables on the spatial distribution of tree species in the Sinharaja FDP. A positive association indicates that the species is more likely to be located in areas of the plot where the value of the environmental variable is high, whereas a negative association indicates that the species is more likely to be located in areas of the plot where the value of the environmental variable is low. (a) Percentage of the 57 study species showing significant associations to a given environmental variable, separately for life stage, (b) same as (a), but only for positive associations, (c) same as (a), but only for negative associations.

For most species, the observed distributions were significantly associated with the parametric intensity functions, which represent the impact of environmental heterogeneity on the species distribution: 46, 51, and 51 out of 57 species showed a significant Berman test for recruits, juveniles, and adults, respectively (Appendix S13). All species and life stages showed at least one significant environmental variable in the intensity model (Appendix S14) and the percentage of species that were associated with a given environmental variable was very similar among the three life stages (Figure 2). For example, the average number of significant variables per species was 4.5, 5.2, and 4.5 for recruits, juveniles, and adults, respectively, and the corresponding average numbers of positive associations were 1.9, 2.6, and 2.2, and that of negative associations were 2.6, 2.6, and 2.3.

Several species showed significant associations with all eight environmental variables, such as recruits of the canopy species *Shorea megistophylla*, juveniles of the subcanopy species *Cryptocarya wightiana*, and adults of the understorey species *Humboldtia laurifolia* (Appendix S14). However, some species were only significantly associated with one or two environmental variables, such as recruits of the understorey species *Garcinia morella* (PC2), juveniles of the subcanopy species *Syzygium cylindricum* (Elevation and PC2), and adults of the understorey species *Actinodaphne albifrons* (Elevation and PC2; Appendix S14).

Elevation and the first principal component (PC1) of the soil variables were the most important environmental variables, which were significantly associated with approximately two-thirds of all species distributions (Figure 2). Curvature was associated with a somewhat lower number of species (approximately 40%), whereas all other variables were associated with roughly 50%–60% of all species (Figure 2a). Elevation, PC1, and the TWI showed more negative than positive species associations, whereas slope showed more positive associations (Figure 2b,c).

### 3.3 Specialist and Generalist Species (Questions ii and iii)

**Figure 3:**
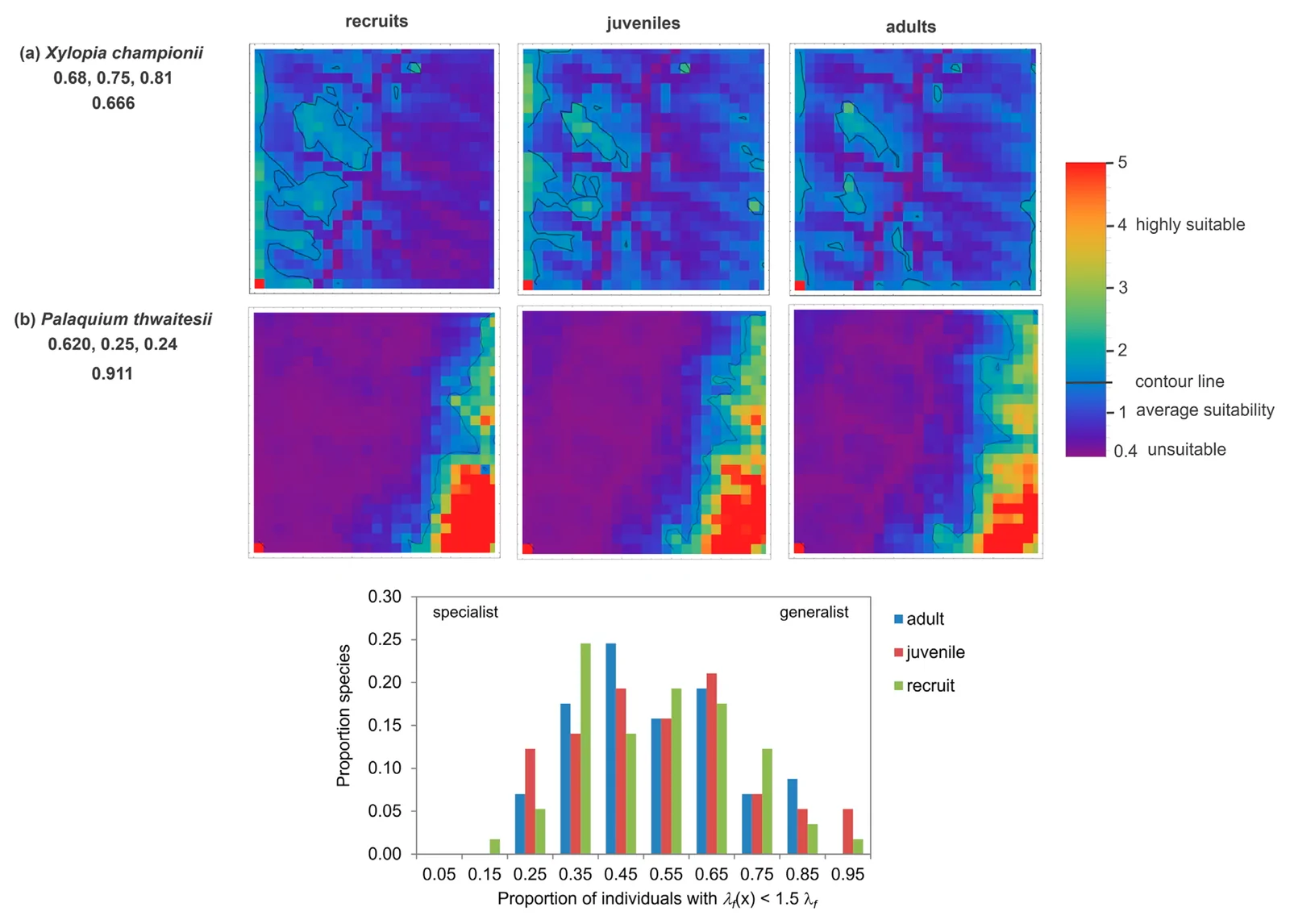
Specialist and generalist species. (a, b) Examples for the normalized intensity functions *λ_f_*(*x*)/*λ_f_* of the generalist species *X. championii* and the specialist species *P. thwaitesii* across life stages. The plot size is 500 × 500 m, and a small quadrat is 20 × 20 m. The black contour line indicates the threshold of *λ_f_*(*x*) = 1.5 *λ_f_*, the numbers below the species names give for recruits, juveniles and adults, respectively, the proportion of individuals predicted by the intensity function to be located in quadrats with *λ_f_*(*x*)/*λ_f_* < 1.5, and the single number below is the correlation coefficient between the intensity functions of recruits and adults. (c) The distribution of the proportion of individuals predicted to be located in quadrats with an intensity *λ_f_*(*x*) < 1.5 *λ_f_*.

Different species often showed quite differently structured intensity functions. For example, *Palaquium thwaitesii* (Figure 3b) showed large areas in the plot with very low intensity (dark blue), but had in the south-east corner a patch with very high intensity that reaches up to five times the average intensity. The intensity function predicts that most individuals of this species would be located in the high intensity areas (where *λ*_*f*_(*x*) > 1.5 *λ*_*f*_).

*Palaquium thwaitesii* is therefore a specialist species that occurs mostly in the high-intensity hotspots (the red areas). In contrast, *Xylopia championii* shows quite flat intensity functions with similar intensity values that rarely exceed 1.5 times the average intensity (Figure 3a). Thus, the intensity function predicts that most individuals would be located in areas with *λ*_*f*_(*x*) < 1.5 *λ*_*f*_. This species is therefore a generalist species that occurs throughout the plot, but without hotspots of high intensity.

We used the proportion of individuals that are predicted by the intensity function to be located in quadrats with an intensity *λ*_*f*_(*x*) < 1.5 *λ*_*f*_ as a measure to locate a species along the specialist–generalist gradient. Figure 3c shows that there were relatively few species classified as strong specialist or generalist species, and most species show rather moderate environmental dependency, with proportions between 35% and 65% of the individuals located in quadrats with *λ*_*f*_(*x*) < 1.5 *λ*_*f*_ (Appendix S7a). There was also relatively little variation among life stages in the distribution of the specialist–generalist properties. Appendix 7b shows that 35% of all species did not have highly suitable quadrats with intensities higher than 2.5 times the mean intensity.

### 3.4 Environmental Dependency Across Life Stages (Question iii)

Figure 3a,b shows examples for two species with little variation between the intensity functions of the different life stages. The intensity functions of conspecific recruits and adults were highly correlated, as indicated by a Pearson correlation coefficient of 0.66 for the generalist species *X. championii* (Figure 3a) and 0.91 for the specialist species *P. thwaitesii* (Figure 3b).

**Figure 4:**
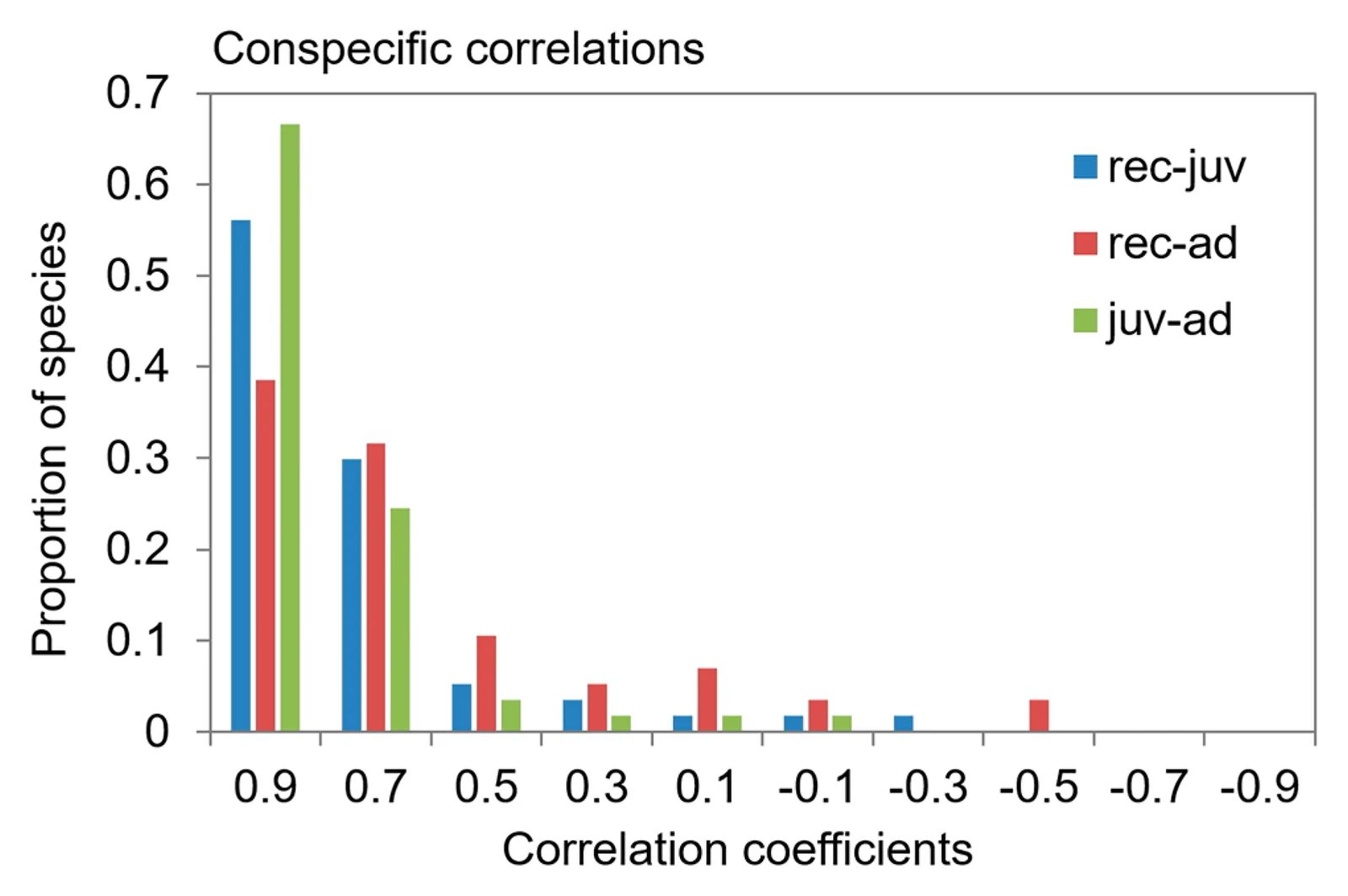
Does environmental dependency change with life stage (Question iii)? The graph shows the distribution of the correlation coefficients between the intensity functions of conspecifics for different life stages. For most species, the environmental dependence did not change with life stages, as indicated by high correlation coefficients between the intensity functions of the different combinations of life stages.

When repeating this analysis for all species, we found that the correlation between the intensity functions of different life stages of conspecifics was generally very high. For example, the correlation between the intensity functions of recruits and juveniles was for 86% of all species larger than 0.6; between juveniles and adults, this occurred for 91% of all species; and between recruits and adults for 70% of all species (Figure 4). Thus, the correlation between the intensity functions of subsequent life stages tended to be somewhat higher than that of recruits and adults, which are separated by a larger time-span.

Additionally, the strength of habitat association, as measured by the proportion of individuals that are predicted by the intensity function to be located in quadrats with low to moderate intensity, correlated very well between life stages (Appendix S15). Nevertheless, one species (with a significant Berman test for recruits) showed a negative association between the adults and recruits intensity functions (*Myristica dactyloides*). *Shorea affinis* and *Mesua nagassarium* showed the highest consistent positive correlation between life stages (Appendix S8).

**Figure 5:**
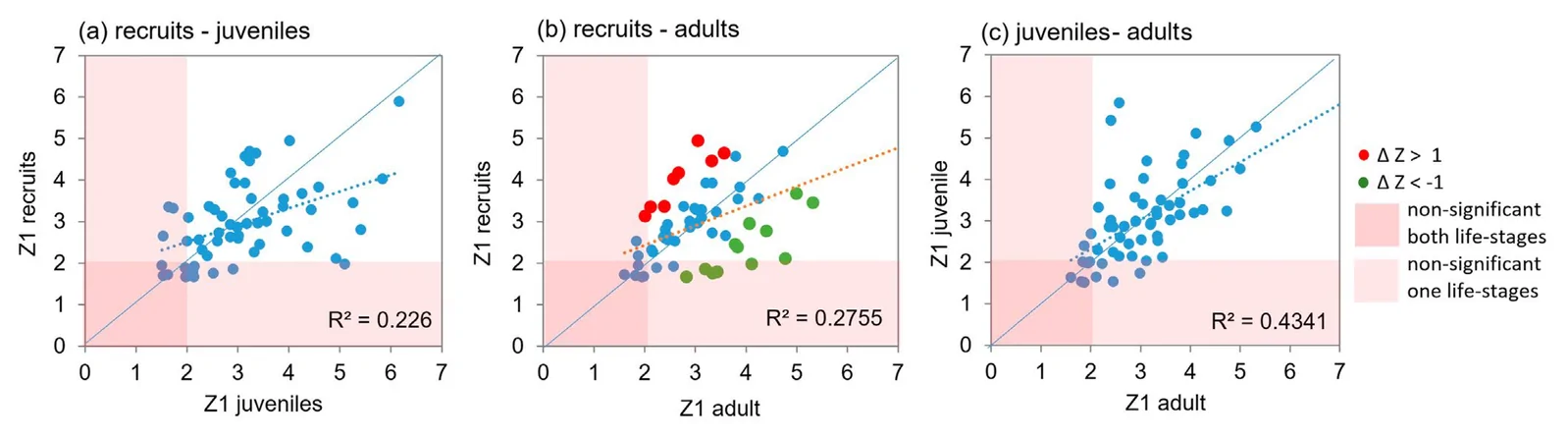
The strength of habitat association, as measured by the *Z*-value of the Berman test, for the different life stage combinations. Each dot represents one species.

Finally, the strength of association of a species pattern to the intensity function, as measured by the *Z*-value of the Berman test, was also positively correlated for all life stage combinations (Figure 5). Most species showed for adults and recruits a similar strength of association between the species distribution and the respective intensity function (Figure 5b), with few species showing stronger association in recruits (red, 8) or stronger association in adults (green, 13).

### 3.5 Similarity or Segregation of the Environmental Dependence Between Species (Questions iii and iv)

**Figure 6:**
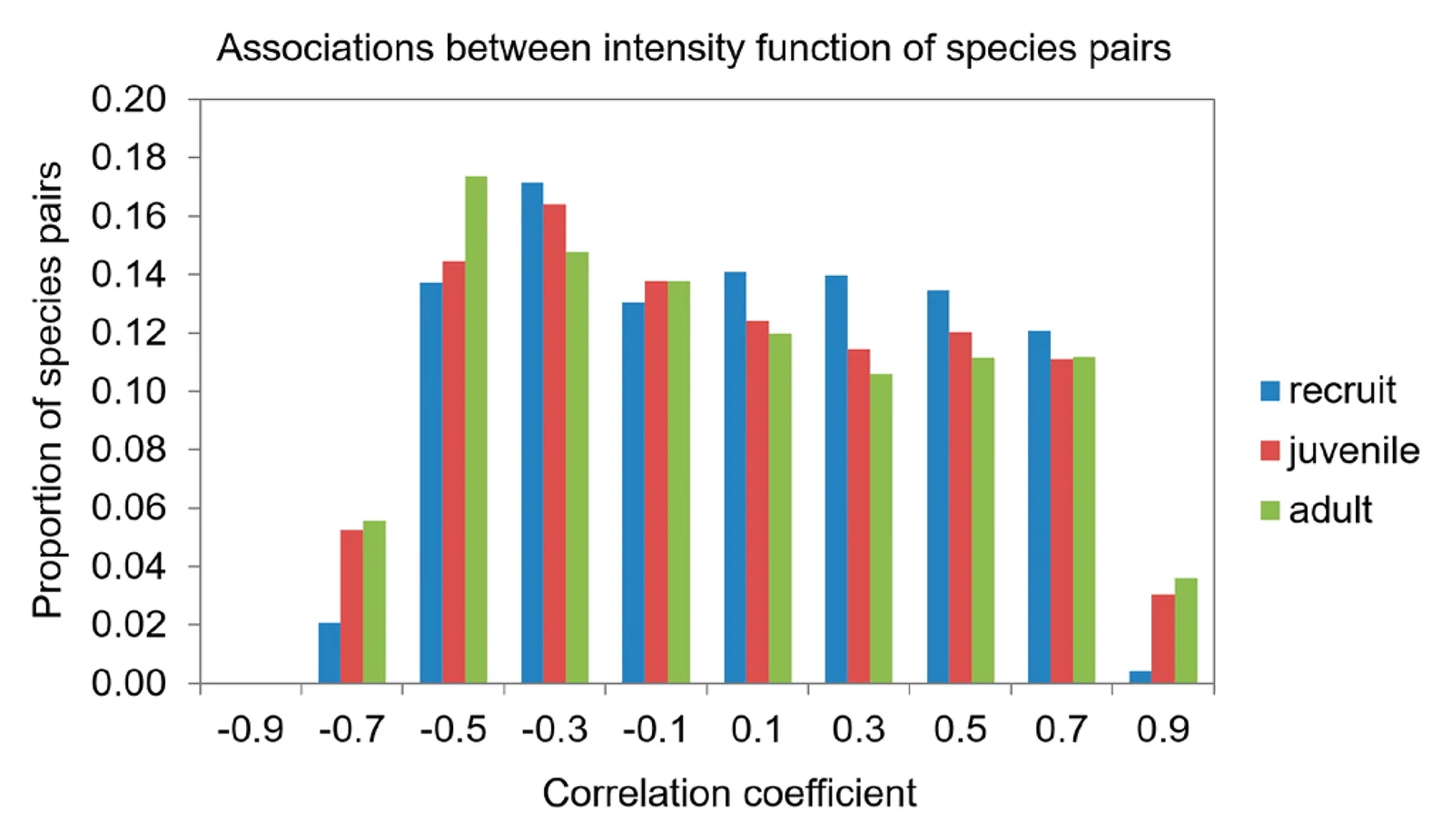
Are the environmental dependencies of different species correlated (Question iv). The distribution of the Pearson correlation coefficients between the intensity functions of all pairs of species, separately for life stage. A high correlation coefficient indicates that the environmental dependence of the two species is very similar, whereas a high negative correlation coefficient indicates that the species use different areas of the plot.

Most species showed weak correlations in their intensity functions: 85%, 80%, and 80% of the recruits, juveniles, and adults showed correlation coefficients larger than −0.6 and smaller than 0.6 (Figure 6), respectively. However, on average, 4% of all species pairs showed strongly opposed habitat associations (i.e., correlation coefficients < −0.6), but the proportion of species pairs with strongly positively correlated intensity functions (i.e., correlation coefficients > 0.6) increased with life stage from 12.5% for recruits to 14% for juveniles and 17% for adults.

## 4 Discussion

Tropical rainforests are among the most diverse and complex ecosystems on the planet and support a high number of tree species (Wright 2002). The spatial distribution of tree species across life stages is influenced by a range of environmental factors, including topographic and edaphic variables (Harms et al. 2001; Gunatilleke et al. 2006; Paoli et al. 2006; John et al. 2007; Allié et al. 2015; Guo et al. 2017; Liu et al. 2018). Here, we used methods of spatial point process theory to examine the influence of such environmental factors on the local distribution (i.e., the spatial intensity function) of different life stages of tree species in a tropical rainforest. We investigated how the proportion of specialist versus generalist species varies among species, if the environmental factors and the strength of environmental dependency changed with life stage, and to what degree the environmental dependencies of species and life stages were correlated.

We found that most species distributions showed significant associations with at least one environmental variable, and all environmental variables contributed to the environmental dependency of a high proportion of species (between 30% and 80%), with elevation and the first principal component of the soil nutrients variables (PC1) being the most important environmental variables. Species distributions were mostly negatively influenced by elevation, PC1, and the TWI, and mostly positively by slope. Additionally, most species showed an intermediate strength of environmental dependency, with relatively few generalist and specialist species. For conspecifics, we found a striking similarity in the intensity functions of different life stages, and the strength of the associations of the species distribution to the intensity function was positively correlated between life stages. Finally, within a given life stage, the intensity functions of different species were only in a few cases strongly and negatively correlated, but the proportion of species pairs with strongly positively correlated intensity functions increased with life stage from 12.5% for recruits to 14% for juveniles and 17% for adults.

### 4.1 Environmental Drivers

All eight environmental variables considered here contributed significantly to the intensity function of a high proportion of all species (usually > 50%), but in quite different mixtures. Given that our variables covered a wide range of possible environmental influences, including topography, wetness, and soil nutrients, this resulted in a wide variety of responses of individual species to the local environment (Appendices S8, S9, and S16). Topography drives the different micro-environmental conditions prevailing within the large elevational range of the Sinharaja FDP (150 m), and previous studies showed that the distribution of species and species assemblages were associated with topography (Gunatilleke et al. 2006; Punchi-Manage et al. 2013). Additionally, topographic features such as slope and aspect influence the distribution of nutrients through geomorphic processes (John et al. 2007; Liu et al. 2018), and topography may influence soil moisture (Sørensen et al. 2006) and the amount of direct and diffuse radiation (Larsen and Speckman 2004). It is therefore not surprising that our approach of fitting a parametric intensity function to the species occurrence data revealed an important contribution of topography. The most important topographic variable was elevation, which is known to produce distinct changes in micro-environmental conditions and forest structure along the elevational gradient in the Sinharaja FDP (Gunatilleke et al. 2006). For example, soil in the high-elevation areas shows lower nutrient levels and is usually drier, shallower, and more prone to desiccation than lower elevation areas (Ashton and Berlyn 1992; Ashton et al. 1995; Gunatilleke et al. 2006; Ediriweera et al. 2008). High elevation areas were preferred by species such as *Palaquium thwaitesii* (Appendix S16), *M. nagassarium*, and *S. disticha* (Appendix S8).

We also found that soil nutrients variables contributed to a similar extent as topographic variables to the intensity function. Thus, there are important aspects of the soil nutrients distribution that are not highly correlated with topographic features, which improved the assessment of the environmental dependency of species. This result is in agreement with a study by Baldeck et al. (2013) where soil and topography accounted in several tropical forest plots for similar variation in local tree community composition, and where adding soil resource variables to topography nearly doubled the inferred role of habitat association.

### 4.2 Strength of Environmental Dependency

We used two independent measures describing the strength of environmental dependency. The first measure describes the structure of the intensity functions, whereas the second measure describes the strength of the association between the observed species distribution and the intensity function. In general, we found only a relatively small proportion of specialist species, indicating that most species at the Sinharaja FDP show moderate associations with environmental features that modulate the intensity function, rather than showing large areas with low suitability. We discuss the consequences of this pattern below in the section “*Correlations in environmental dependencies among species*.”

Because plants can only move during the seed stage, seed dispersal defines the initial template for tree distribution, which is then subject to the subsequent effects of the local environment and density-dependent processes (Webb and Peart 2000; Shen et al. 2009; Kanagaraj et al. 2011). Thus, the distribution of species is not only influenced by the underlying environmental association, as described by the intensity function, but also by additional spatially structured mechanisms unrelated to the environment. For example, dispersal limitation may cause locally higher densities of the focal species that are unrelated to the environmental heterogeneity. Additionally, our study design cannot rule out the possibility that other biotic processes, such as competition or facilitation, contribute to the spatial distribution of tree individuals. Our second measure of the strength of environmental dependency considers such effects by relating the observed species distribution to the intensity function. Indeed, as expected, the majority of species distributions showed a significant positive association with their corresponding intensity function (80%–90%), but again, most species showed a rather moderate strength of association and only a few species (10%–20%) showed high *Z*-values (> 4; Appendix S7c). This result explains earlier findings of Punchi-Manage et al. (2013). They determined groups of species with similar associations to topographic variables (i.e., species assemblages) and investigated the degree to which the different species assemblages were internally homogeneous but different from other assemblages. They found that topographically driven species assemblages existed at the Sinharaja FDP, but they were weak, as only a few species were confined to a single assemblage (Punchi-Manage et al. 2013). Thus, stochastic effects or spatially structured mechanisms such as dispersal also play a prominent role in structuring this forest.

### 4.3 Changes With Life Stage

We conducted a variety of analyses that all indicated that for most species, the strength and drivers of environmental dependency were very similar across life stages (Figures 4 and 5). The maintenance of similar intensity functions across life stages explains the earlier result by Punchi-Manage et al. (2013) that species assemblages did not change with life stage. However, this result is in contrast to other studies in forest communities that indicated important shifts in habitat association with life stages (e.g., Webb and Peart 2000; Paoli et al. 2006; Comita et al. 2007; Lai et al. 2009; Kanagaraj et al. 2011; Liu et al. 2018). For example, Lai et al. (2009) found that most habitat associations of species were consistent from the sapling to the juvenile stage but changed at the adult stage. The differences to other studies may be partly explained by different definitions of life stages and corresponding thresholds. For example, Punchi-Manage et al. (2013) used for separation of saplings and adults a dbh threshold of 10 cm, which differed from our definition.

There are mutually non-exclusive mechanisms that may lead to consistent environmental dependencies of species across life stages as observed in the Sinharaja FDP. First, it is likely that a stronger and more consistent habitat association of species across life stages can be found in more environmentally heterogeneous plots. A strong topographic structure of the plot (Figure 1c; 150 m elevation difference) creates a wider range of micro-environmental conditions for species to specialize compared to plots with weak topographic structuring such as Barro Colorado Island (BCI, with a 25-m difference in elevation), where inconsistent environmental dependencies were observed among life stages (Comita et al. 2007; Kanagaraj et al. 2011). The three species with the highest correlation between the intensity functions of adults and recruits in our analysis were all canopy species (*M. nagassarium, S. affinis*, and *S. disticha*) and were strongly associated with the high elevation habitats (Appendix S8) that showed lower nutrient levels and are usually drier and shallower than lower elevation areas. Similarly, in the temperate Fenglin plot, some mid-tolerant tree and shrub species were consistently associated with sunny habitats (Liu et al. 2018). Second, shade-tolerant species may allow their saplings and juveniles to establish and survive in the understory, as suspected for 12 out of 60 species at the subtropical Gutianshan plot that showed consistent positive associations across life stages with the low valley habitat characterized by poor sunlight conditions resulting from a higher density of large trees (Lai et al. 2009). Third, dispersal limitation will cause the majority of seeds and recruiting individuals to be placed not too far from conspecific adults (Zhu et al. 2013). This may be especially the case for seeds dispersed by wind, gravity, gyration, or autochory. Indeed, the three species with the highest correlation between the intensity functions of adults and recruits were all dispersed by autochory (*M. nagassarium*) and anemochory (*S. affinis, S. disticha*), but among the nine species with correlation coefficients > 0.9, four species are dispersed by zoochory (*Mangifera zeylanica, Palaquium thwaitesii, Syzygium neesianum*, and *Nothopegia beddome*i; Appendix S8).

### 4.4 Correlations in Environmental Dependencies Among Species

Niche theory explains the coexistence of species by niche partitioning, where competing species use the environment differently (Hutchinson 1959; Chase and Leibold 2003). The parametric intensity function represents in our analysis the use of the environment by the species. It is therefore of special interest to analyze the distribution of the correlation coefficients of the intensity functions of all pairs of species. Niche theory lets us expect few species with highly correlated intensity functions. Indeed, we found that very few species pairs (86 out of 1596) showed high correlations (> 0.8), and their proportion increased slightly with life stage from 12.5% for recruits to 14% for juveniles and 17% for adults. For example, for the adult stage, we identified a group of 11 species (*Calophyllum thwaitesii, Durio rosayroana, Gyrinops walla, Hopea jucunda, M. nagassarium, P. petiolare, P. thwaitesii, S. affinis, S. trapezifolia, S. disticha*, and *Strombosia nana*) that share a strong association with high elevation areas. Such positive correlations lead to one of the habitat-driven species assemblages observed by Punchi-Manage et al. (2013). Similarly, only a few species pairs showed strong negative correlations in their intensity functions, and the proportion of species pairs showing negative associations increased slightly from 46% for recruits to 50% for juveniles and 52% for adults.

Overall, however, the majority of species pairs showed only weak or no correlations in their intensity functions, which indicates that the environmental conditions where they occur are often independent or characterized by tendencies of avoidance or slight co-occurrence. Thus, most species at the Sinharaja FDP show some differences in their use of the available environmental conditions. This leads to a particular geometric structure where the suitable area of species comprises a mosaic of areas where a given focal species overlaps more strongly with different heterospecifics. Point pattern analysis of the spatial associations of the species distributions, which were sensitive to effects of environmental associations and species interactions, showed similar results: only a small percentage of all species pairs (6%) showed an overall positive association (i.e., the area occupied by a focal species was largely overlapped by the area occupied by another species), and all other species pairs showed either partial overlap (≈40%) or segregation (≈53%) (Wiegand et al. 2007). Thus, most of the species at Sinharaja do not come consistently close to each other, which may lead to a “dilution” of directed pairwise species interactions, a mechanism that has been hypothesized to contribute to species coexistence (Hubbell and Foster 1986; Hubbell 2006; Wiegand et al. 2012; Wang et al. 2016).

## 5 Conclusions

Overall, our study provides valuable insights into the topographic and soil factors that influence tree species composition and intensity in tropical rainforests, particularly in tropical forests with high variability in topography and soil characteristics.

By examining the effects of environmental variables on the intensity of tree species at different life stages, our study provides a nuanced understanding of the environmental dependencies that shape tropical rainforest ecosystems at local spatial scales and could explain the findings of earlier studies. Our findings have important implications for understanding the mechanisms underlying the structure of the mixed-dipterocarp forest in Sri Lanka and highlight the effects of environmental heterogeneity on tropical woody plant diversity.

## Supplementary Material

Additional supporting information can be found online in the Supporting Information section.

Appendices

## Data Availability

Topographic data, first three principal components of soil data for all 20 × 20 m quadrats, and vegetation data (life stages: recruits, juveniles, and adults) in Sinharaja FDP are publicly available (Jayasinghe 2024) at https://doi.org/10.5281/zenodo.14504217.
