# Supplementary material for: Species–Habitat Associations in a Sri Lankan Dipterocarp Forest
Source: J Veg Sci. Author manuscript; Available in PMC 2026 Oct 17. (PMC7619593; doi:10.1111/jvs.70049)
Supplement: Supplementary file 1 [file EMS217228-supplement-Appendices.docx]

**Supporting Information**

**Species-habitat associations in a Sri Lankan dipterocarp forest**

This file contains Appendix S1-S16

1

**Supporting information to the paper**

Species-habitat associations in a Sri Lankan dipterocarp forest. *Journal of Vegetation Science*.

**Appendix S1.** List of species used in our analyses and the number of juveniles, recruits, and adults. C: canopy, SC: subcanopy, US: understorey.

2

**Species	Number of	Number of	Number of**

**Genus	SpeciesName	Stratum	Dispersal Mode**

**code	juveniles	recruits	adults**

ANISCI	*Anisophyllea	cinnamomoides*	C	Zoochory	1343	578	429

BHESCE	*Bhesa	ceylanica*	C	Zoochory	817	195	178

DYSOPE	*Dysoxylum	peerisi*	C	Zoochory	172	60	70

MANGZE	*Mangifera	zeylanica*	C	Zoochory	1017	266	292

MESUFE	*Mesua	ferrea*	C	Autochory	4843	1631	1568

MESUNA	*Mesua	nagassarium*	C	Autochory	13410	6876	3143

SHORAF	*Shorea	affinis*	C	Anemochory	3519	1574	1350

SHORCN	*Shorea	congestiflora*	C	Anemochory	798	629	201

SHORDI	*Shorea	disticha*	C	Anemochory	7276	4041	1424

SHORME	*Shorea	megistophylla*	C	Anemochory	2815	1627	774

SHORST	*Shorea	stipularis*	C	Anemochory	862	459	251

SHORTR	*Shorea	trapezifolia*	C	Anemochory	2420	3542	845

SHORWO	*Shorea	worthingtonii*	C	Anemochory	3848	1302	1277

CALOBR	*Calophyllum	bracteatum*	SC	Zoochory	418	166	129

CALOTH	*Calophyllum	thwaitesii*	SC	Zoochory	1284	555	334

CRYPWI	*Cryptocarya	wightiana*	SC	Zoochory	1363	1313	151

CULLCE	*Durio	ceylanica*	SC	Zoochory	2271	1151	1938

CULLRO	*Durio	rosayroana*	SC	Zoochory	1673	383	876

3

DIOSQU	*Diospyros	quaesita*	SC	Zoochory	191	84	60

GLENUN	*Glenniea	unijuga*	SC	Zoochory	542	209	97

HYDNOC	*Hydnocarpus	octandra*	SC	Autochory	737	73	248

MASTTE	*Mastixia	tetranda*	SC	Zoochory	997	453	281

MYRIDA	*Myristica	dactyloides*	SC	Zoochory	1845	660	1067

PALACA	*Palaquium	canaliculatum*	SC	Zoochory	2452	1329	1669

PALAPE	*Palaquium	petiolare*	SC	Zoochory	3274	3437	484

PALATH	*Palaquium	thwaitesii*	SC	Zoochory	2302	1010	481

PUTRRO	*Putranjiva	roxburghii*	SC	Zoochory	244	56	122

SEMEGA	*Semecarpus	gardneri*	SC	Zoochory	887	616	721

SEMEWA	*Semecarpus	walkeri*	SC	Zoochory	1851	969	684

SHORCR	*Shorea	cordifolia*	SC	Anemochory	3452	2066	999

SYZYCY	*Syzygium	cylindricum*	SC	Zoochory	122	93	98

SYZYMA	*Syzygium	makul*	SC	Zoochory	372	177	95

SYZYNE	*Syzygium	neesianum*	SC	Zoochory	1321	1629	444

SYZYSY	*Syzygium	sylvestre*	SC	Zoochory	202	64	89

ACTIAL	*Actinodaphne	albifrons*	US	219	74	148

CINNCA	*Cinnamomum	capuru-coronde*	US	Zoochory	528	179	226

CINNDU	*Cinnamomum	dubium*	US	Zoochory	90	115	59

CINNVE	*Cinnamomum	verum*	US	Zoochory	248	74	165

DIOSAC	*Diospyros	acuminata*	US	Zoochory	980	563	916

DIOSHI	*Diospyros	hirsuta*	US	Zoochory	491	118	496

ELAESU	*Elaeocarpus	subvillosus*	US	Zoochory	262	193	181

GARCHE	*Garcinia	hermonii*	US	Zoochory	3709	951	4698

GARCMO	*Garcinia	morella*	US	Zoochory	315	54	415

4

GOMPSE	*Ouratea	serrata*	US	Zoochory	249	73	340

GYRIWA	*Gyrinops	walla*	US	Zoochory	204	82	172

HOPEJU	*Hopea	jucunda*	US	Anemochory	753	522	333

HUMBLA	*Humboldtia	laurifolia*	US	Autochory	7517	7369	20732

NARGMA	*Nargedia	macrocarpa*	US	Zoochory	569	111	305

NOTHBE	*Nothopegia	beddomei*	US	Zoochory	414	146	240

SEMESU	*Semecarpus	subpeltata*	US	Zoochory	303	93	153

STRONA	*Strombosia	nana*	US	Zoochory	117	70	239

XYLOCH	*Xylopia	championii*	US	Zoochory	1131	834	1479

EUPHLO	*Litchi	longana*	Other	Zoochory	440	242	100

PODATH	*Podadenia	thwaitesii*	Other	Zoochory	309	137	245

SYZYLI	*Syzygium	lissophyllum*	Other	Zoochory	258	166	55

SYZYWI	*Syzygium	wightiana*	Other	Zoochory	588	209	223

TRICER	*Tricalysia	erythrospora*	Other	Zoochory	287	150	309

**Supporting information to the paper**

Species-habitat associations in a Sri Lankan dipterocarp forest. *Journal of Vegetation Science*.

**Appendix S2.** Descriptive statistics of the environmental variables used.

5

**Variable name**

**Abbreviation**

**Mean**

**SD**

**Minimum**

**Maximum**

**Soil measures**

Aluminium (mg kg^-1^) Calcium (mg kg^-1^) Iron (mg kg^-1^) Potassium (mg kg^-1^) Magnesium (mg kg^-1^) Manganese (mg kg^-1^) Sodium (mg kg^-1^) Soil pH (water)

Soil pH (CaCl2) Soil pH (BaCl2)

Phosphate determined by extraction with Bray-1 solution (mg kg^-1^)

Total exchangeable bases (cmolc kg^-1^,

i.e centimoles of charge per kg) Effective cation exchange capacity (cmolc kg^-1^)

Base saturation (TEB / ECEC * 100)

RB-NH4 bags (mg kg^-1^) RB-NO3 bags (mg kg^-1^) RB-PO4 bags (mg kg^-1^) Principal component 1

Principal component 2

Principal component 3

Al	132.14	42.50	42.18	311.05

Ca	73.50	29.19	26.59	214.08

Fe	2.04	1.03	0.303	8.25

K	26.39	5.49	16.28	45.15

Mg	21.90	4.05	13.70	52.21

Mn	2.36	0.93	0.91	6.59

Na	4.61	2.79	0.04	17.99

pH1	4.95	0.19	4.43	5.42

pH2	3.97	0.09	3.71	4.16

pH3	4.07	0.09	3.72	4.35

Bray-P	2.38	0.81	0.72	3.93

TEB	0.63	0.02	0.36	1.46

ECEC	2.12	0.49	1.177	4.17

BS	30.89	8.89	13.37	67.74

RB-NH4	4.05	3.09	0.26	43.77

RB-NO3	41.19	12.03	17.40	70.88

RB-PO4	0.07	0.04	0.01	0.35

PC1	0	2.68	-5.32	8.61

PC2	0	2.01	-3.79	10.65

PC3	0	1.42	-6.07	5.25

**Topographic variables**

Elevation (m) Slope (°) Aspect (°) Curvature

Topographic wetness index

Altitude above channels (m)

-	470.2	33.89	425.3	574.4

-	21.62	8.01	0.64	38.13

-	206.51	98.5	0.56	359.5

-	-0.01	2.41	-0.8	7.34

TWI	4.98	1.57	3.09	11.59

ACH (m)	7.27	8.04	0	37.96

**Supporting information to the paper**

Species-habitat associations in a Sri Lankan dipterocarp forest. *Journal of Vegetation Science*.

**Appendix S3.** Description of topographic variables

Elevation (m) was calculated by taking the average of the four corners of the 20 × 20 m^2^ quadrat. The slope (^0^) was the mean angular departure from the horizontal of each of the four triangular planes generated by joining three of the four corners of the quadrat and aspect was the compass direction a slope faces. The rate of change of slope was defined as the curvature: positive values indicated a convex surface whereas negative values indicated a concave surface. To capture topographic wetness, we used the two indices TWI and ACH. TWI is the ratio of each quadrat's area upslope to that quadrat's local slope and ACH is the vertical distance from the channel network calculated by estimating catchment

area and channel network using elevation data.

6

**Supporting information to the paper**

Species-habitat associations in a Sri Lankan dipterocarp forest. *Journal of Vegetation Science*.

**Appendix S4. Correlations between environmental variables**

A) Correlation matrix for the 17 soil variables. Bold values indicate significant correlation coefficients.

7

Soil measure

Al

Ca

Fe

K

Mg

Mn

Na

pH1

pH2

pH3

Bray-P

TEB

ECEC

BS

RB- NH4

RB- NO3

RB- PO4

Al

1

**-0.307**	1

**0.677	-0.269**	1

**0.534	0.169	0.462**	1

**0.392	0.528	0.231	0.678**	1

-0.017	**0.577**	-0.077	**0.507	0.651**	1

**0.635	-0.099	0.387	0.363	0.547	0.193**	1

**-0.747	0.264	-0.541	-0.373	-0.163	0.119	-0.353**	1

**-0.811	0.361	-0.724	-0.528	-0.336**	0.062	**-0.546	0.716**	1

**-0.839	0.319	-0.687	-0.497	-0.238	0.114	-0.365	0.783	0.773**	1

**0.230	-0.310	0.400	0.509**	-0.075	**-0.135	-0.183	-0.215	-0.341	-0.402**	1

**-0.105	0.960	-0.138	0.383	0.713	0.695	0.112	0.135	0.164	0.173	-0.265**	1

**0.942**	0.023	**0.617	0.656	0.625	0.224	0.660	-0.685	-0.740	-0.761	0.138	0.235**	1

**-0.780	0.793	-0.569	-0.165	0.127	0.420	-0.394	0.631	0.712	0.733	-0.308	0.668	-0.536**	1

**0.446**	-0.077	**0.327	0.450	0.389	0.283	0.440	-0.280	-0.428	-0.331	0.105	0.096	0.472	-0.250**	1

**0.412	-0.094	0.498	0.580	0.196	0.137	0.109	-0.473	-0.572	-0.508	0.627**	0.017	**0.413	-0.296	0.316**	1

**0.226	-0.323	0.452	0.376**	-0.001	**-0.165**	-0.072	**-0.156	-0.443	-0.370	0.714	-0.267	0.133	-0.334	0.255	0.503**	1

Ca

Fe

K

Mg

Mn

Na

pH1

pH2

pH3

Bray-P

TEB

ECEC

BS

RB-NH4

RB-NO3

RB-PO4

B) Correlation matrix for nine environmental variables (Elevation, Slope, Aspect, Curvature, TWI, ACH, PC1, PC2, PC3). Bold values indicate

significant correlation coefficients.

8

Covariates

ACH

TWI

Slope

Elevation

Curvature

Aspect

PC1

PC2

PC3

ACH

1	.	.	.	.	.	.	.	.

**-0.502**	1	.	.	.	.	.	.	.

**-0.092	-0.473**	1	.	.	.	.	.	.

**0.277	-0.453	0.433**	1	.	.	.	.	.

**0.569	-0.544**	0.074	**0.304**	1	.	.	.	.

**-0.192**	-0.007	**0.314	0.390	0.020**	1	.	.	.

**0.426	-0.179	-0.294	-0.138	0.143	-0.625**	1	.	.

**-0.097**	-0.062	**0.134	0.219**	-0.017	0.034	8.56e-13	1	.

**0.322	-0.387	0.313	0.779	0.264	0.377**	1.07e-11	-4.33e-12	1

TWI

Slope

Elevation

Curvature

Aspect

PC1

PC2

PC3

**Supporting information to the paper**

Species-habitat associations in a Sri Lankan dipterocarp forest. *Journal of Vegetation Science*.

**Appendix S5.** Methodological details of Berman test

We used the Berman test (Berman 1986; Wiegand and Moloney 2014) to determine the strength of association of the observed species pattern to the estimated intensity function. The test is performed by comparing the observed distribution of *λ*(*x*) taken at the locations *x*i of the individuals *i* of the given species and the predicted distribution arising under a suitable null model that randomizes the individuals of the species. We use here the toroidal shift null model, as already proposed by Berman (1986), to conserve the observed spatial autocorrelation (aggregation) pattern of the species locations in good approximation (Wiegand and Moloney 2014). This is important because ignoring species aggregation not related to environmental variables (e.g., caused by dispersal limitation or clumped animal seed dispersal) may lead to an overestimation of the significance of the log-linear regression model. The test

statistic (the z-score)

*Z* = (*S* - *μ*)/*σ*

introduced by Berman (1986) is based on the sum *S* of the intensity values *λ*(*xi*) at the locations x*i* of the species. The value *μ* is the predicted mean value of *S* under the 199 realizations of the null model and *σ*^2^ is the corresponding variance*.* The null distribution of this test statistic is approximately the standard normal distribution. We assessed significant deviation of the *Z* values from the null model at α = 0.05. The Berman test was conducted with the software Programita ([http://programita.org,](http://programita.org/) Wiegand and

Moloney 2014).

**Reference**

Berman, M. 1986. “Testing for spatial association between a point process and another stochastic process”. *Applied Statistics*, 35:54–62. https://doi.org/10.2307/2347865

Wiegand, T. and Moloney, K. A. 2014. *Handbook of Spatial Point- Pattern Analysis in Ecology*.

Chapman & Hall/CRC.

9

**Supporting information to the paper**

Species-habitat associations in a Sri Lankan dipterocarp forest. *Journal of Vegetation Science*.

**Appendix S6.** Method of allocating a given species within the specialist-generalist gradient

A specialist species shows a high intensity function in only a few sites, whereas a generalist species shows average intensities in most of the area. To find out where a focal species *f* is located at the generalist-specialist axis, we estimated the proportion of individuals predicted by the intensity function to be placed at locations of low to moderate environmental suitability. More technically, in this analysis we assume that the distribution of the species follows a heterogeneous Poisson process driven by the intensity function *λf*(*x*) (Wiegand & Moloney, 2014). That means that the individuals are distributed over the quadrats x proportionally to the value of *λf*(*x*). We estimated the proportion of individuals located in quadrats *x* with *λf*(*x*) < *c λf*, where *λf* is the average of the intensity function over all quadrats of the plot and *c* a factor. After plotting the intensity functions (Appendix S16, S8, S9), we found that the factors *c*

= 1.5 and 2.5 were suitable to allocate species at the generalist-specialist axis. With *f* = 1.5, a high proportion of individuals of a generalist species would be located in areas of not very high intensity (i.e., *λf*(*x*) < 1.5 *λf*). Note that a generalist species would show in most quadrats a normalized intensity function *λf*(*x*)/*λf* close to one (i.e., the intensity is similar to the average intensity) and therefore rarely reaches above the 1.5 *λf* threshold. Conversely, a high proportion of individuals of a specialist species would be located in areas of a high intensity functions (i.e., quadrats of high habitat suitability) and consequently only few would show an intensity *λf*(*x*) < 1.5 *λf* . When using factor 2.5 we can even better determine the specialist species. The distribution of this proportion over species then provides us with

information on degree of specialist, intermediate and generalist species.

10

**Supporting information to the paper**

Species-habitat associations in a Sri Lankan dipterocarp forest. *Journal of Vegetation Science*.

**Appendix S7.** The distribution of the strength of habitat association among species. In a) and

b) the strength of habitat association was measured by the proportion of individuals located in

quadrats with an intensity *λf*(*x*) < 1.5 *λf* (a) or < 2.5 *λf*

(b) if the intensity completely drove the

species distribution. A low proportion means that the species is located in few very high quality quadrats with high intensity (i.e., a specialist species), whereas high proportion means that the species is located mainly in average quality quadrats (i.e., a generalist species). (c) The strength of association of the species pattern to the intensity function, as measured by the Z-score of the Berman test. Z-values below 1.95 are non-significant at α = 0.05 and larger Z- values mean stronger association.

11

**Supporting information to the paper**

Species-habitat associations in a Sri Lankan dipterocarp forest. *Journal of Vegetation Science*.

**Appendix S8.** The nine species with the strongest correlation between the intensity function of recruits, and adults, with correlation coefficients above 0.9. The three numbers indicate the proportion of individuals of recruits, juveniles, and adults predicted to be located in quadrats *x* with a normalized intensity λ*f*(x)/λ*f* < 1.5, respectively, and the single number below is the correlation coefficient between the intensity functions of recruits and adults. Other conventions as in Appendix S7.

(a) *Mesua nagassarium*

(b) *Shorea affinis*

(c) *Shorea disticha*

0.398

0.407

0.970

0.417

0.376

0.313

0.948

0.319

0.267

0.258

0.932

0.266

(d) *Mangifera zeylanica*

(e)*Palaquium thwaitesii*

(f) *Humboldtia laurifolia*

12

0.528

0.687

0.923

0.716

0.199

0.204

0.911

0.239

0.515

0.522

0.910

0.615

(g) *Mesua ferrea*

(i) *Nothopegia beddomei*

(h) *Syzygium neesianum*

0.35

0.4

0.909

0.43

0.491

0.519

0.903

0.528

0.313

0.460

0.902

0.416

13

**Supporting information to the paper**

Species-habitat associations in a Sri Lankan dipterocarp forest. *Journal of Vegetation Science*.

**Appendix S9.** The six species with the lowest correlation between the intensity function of recruits and adults. The three numbers below the lower panel indicate the proportion of individuals of recruits, juveniles, and adults predicted to be located in quadrats *x* with

normalized intensity λ*f*(x)/λ*f*

< 1.5, respectively, and the single number below is the

correlation coefficient between the intensity functions of recruits and adults. Other conventions as in Appendix S7.

(a) *Bhesa ceylanica*

(b) *Cinnamomum dubium*

(c) *Shorea congestiflora*

0.789

0.591

0.003

0.617

0.686

0.693

-0.126

0.751

0.757

0.844

0.03

0.851

(d) *Syzygium makul*

(e) *Myristica dactyloides*

(f) *Cinnamomum capuru-coronde*

14

0.853

0.400

-0.129

0.850

0.766

0.870

-0.430

0.886

0.746

0.910

-0.561

0.843

15

**Supporting information to the paper**

Species-habitat associations in a Sri Lankan dipterocarp forest. *Journal of Vegetation Science*.

**Appendix S10.** Maps of 25-ha plot for aspect, curvature, topographic wetness index, and three principal components for soil variables**.** The six environmental variables shown here together with the two shown in Figure 1 were used to determine the intensity functions of individual species

16

**Supporting information to the paper**

Species-habitat associations in a Sri Lankan dipterocarp forest. *Journal of Vegetation Science*.

**Appendix S11.** Principal component analysis of the soil variables. Shown are the loadings of

the first three components, their eigenvalues, and the % of variance explained.

17

**Soil variable	PC1	PC2	PC3**

Al	0.344	0.017	0.203

Ca	-0.129	0.42	-0.09

Fe	0.295	-0.0365	-0.053

K	0.249	0.251	-0.265

Mg	0.146	0.417	0.032

Mn	0.003	0.413	-0.099

Na	0.212	0.154	0.172

pH1	-0.288	0.042	-0.137

pH2	-0.34	0.026	-0.042

pH3	-0.329	0.053	-0.035

Bray-P	0.173	-0.139	-0.533

TEB	-0.05	0.473	-0.064

ECEC	0.32	0.177	0.175

BS	-0.283	0.27	-0.189

RB-NH4	0.197	0.133	0.009

RB-NO3	0.238	0.0251	-0.375

RB-PO4	0.177	-0.138	-0.459

Eigenvalues	7.718	4.029	2.022

Variance (%)	36.6	23.3	17.9

Cum. Variance (%)	36.6	59.9	77.8

**Supporting information to the paper**

Species-habitat associations in a Sri Lankan dipterocarp forest. *Journal of Vegetation Science*.

**Appendix S12.** The loadings of the 17 soil variables for the first two principal components (Appendix S11). We find a clear separation into the 3 groups. A first group with large negative values of PC1 is mostly associated with negative loadings of pH and base saturation in PC1, a second group with high values of PC1 is associated with high positive loadings of Al, ECEC, Fe, K, RB-No3 and Na, and a third group with high values of PC2 shows high positive loading of Ca, TEB; Mn and Mg.

18

**Supporting information to the paper**

Species-habitat associations in a Sri Lankan dipterocarp forest. *Journal of Vegetation Science*.

**Appendix S13.** Results of the Berman test to test for a positive association between the intensity function and the locations of the species. Shown is the Z-score for the different species and life stages, and the corresponding P-value. Z- values < 1.95 (marked grey)

indicate a non-significant association with α = 0.05.

19

**Species**

**Z adults**

**P adults**

**Z juveniles**

**P juveniles**

**Z recruits**

**P recruits**

ACTIAL	3.123	0.0018	2.033	0.0424	3.094	0.002

ANISCI	2.013	0.0444	2.69	0.0074	3.125	0.0018

BHESCE	3.576	0.0004	3.363	0.0008	4.645	0.0001

CALOBR	3.044	0.0024	3.398	0.0007	2.959	0.0032

CALOTH	3.125	0.0018	4.44	0.0001	3.283	0.001

CINNCA	3.2	0.0014	2.91	0.0036	0.0658

CINNDU	4.12	0.0001	5.104	0.0001	1.972	0.0488

CINNVE	2.456	0.0143	2.243	0.0251	2.565	0.0105

CRYPWI	5	0.0001	4.258	0.0001	3.676	0.0002

CULLCE	2.235	0.0257	1.966	0.05	0.0601

CULLRO	3.795	0.0002	3.141	0.0017	4.565	0.0001

DIOSAC	0.0658	2.007	0.0455	2.529	0.0117

DIOSHI	0.0703	0.126	0.091

DIOSQU	0.0615	0.131	0.0524

DYSOPE	3.323	0.0009	3.23	0.0012	4.456	0.0001

ELAESU	3.014	0.0026	2.546	0.0111	3.278	0.0011

EUPHLO	7.681	0.0001	6.167	0.0001	5.892	0.0001

GARCHE	2.57	0.0102	5.836	0.0001	4.03	0.0001

GARCMO	3.418	0.0006	3.494	0.0005	3.225	0.0013

GLENUN	2.904	0.0037	2.993	0.0028	2.861	0.0042

GOMPSE	4.41	0.0001	3.967	0.0001	2.775	0.0056

GYRIWA	3.059	0.0023	4.021	0.0001	4.943	0.0001

HOPEJU	2.572	0.0102	2.148	0.0324	0.0549

HUMBLA	3.337	0.0009	2.628	0.0088	2.725	0.0065

HYDNOC	0.1096	0.1031	0.0854

MANGZE	0.0615	2.4	0.0168	2.17	0.03

MASTTE	2.458	0.0143	0.126	2.649	0.0083

MESUFE	3.6	0.0003	3.02	0.0025	2.663	0.0078

MESUNA	2.597	0.0096	2.605	0.0093	2.532	0.0114

MYRIDA	2.889	0.004	3.567	0.0004	3.006	0.0027

NARGMA	2.388	0.0173	3.888	0.0001	3.363	0.0008

1.533

1.722

1.631

1.602

1.873

1.923

1.697

1.943

1.536

1.511

1.841

1.817

1.871

1.887

1.846

20

NOTHBE	4.075	0.0001	3.183	0.0015	2.948	0.0033

PALACA	1.981	0.0477	2.007	0.0455	0.0949

PALAPE	0.0524	1.978	0.0488	0.0969

PALATH	2.675	0.0076	2.864	0.0042	4.175	0.0001

PODATH	2.994	0.0028	0.0836	3.316	0.0009

PUTRRO	3.876	0.0001	4.585	0.0001	3.825	0.0001

SEMEGA	4.733	0.0001	3.234	0.0012	4.689	0.0001

SEMESU	2.116	0.0349	0.101	3.346	0.0008

SEMEWA	2.77	0.0056	2.443	0.0147	3.359	0.0008

SHORAF	2.456	0.0143	2.851	0.0044	2.928	0.0035

SHORCN	3.794	0.0002	3.435	0.0006	2.449	0.0147

SHORCR	2.14	0.0324	2.303	0.0214	2.32	0.0209

SHORDI	2.375	0.0178	2.854	0.0044	2.629	0.0088

SHORME	3.334	0.0009	3.143	0.0017	3.926	0.0001

SHORST	4.253	0.0001	3.268	0.0011	3.548	0.0004

SHORTR	3.208	0.0014	2.944	0.0033	3.929	0.0001

SHORWO	3.834	0.0001	4.369	0.0001	2.384	0.0173

STRONA	2.162	0.0308	3.326	0.0009	2.26	0.0244

SYZYCY	4.778	0.0001	4.928	0.0001	2.101	0.0357

SYZYLI	3.845	0.0001	3.903	0.0001	3.534	0.0004

SYZYMA	2.832	0.0047	2.141	0.0324	0.0969

SYZYNE	2.407	0.0164	3.006	0.0027	2.599	0.0096

SYZYSY	3.347	0.0008	2.523	0.0117	0.0801

SYZYWI	3.438	0.0006	2.12	0.0349	0.0751

TRICER	2.406	0.0164	5.413	0.0001	2.805	0.0051

XYLOCH	5.326	0.0001	5.266	0.0001	3.448	0.0006

1.753

1.783

1.665

1.641

1.734

1.947

1.673

1.661

**Supporting information to the paper**

Species-habitat associations in a Sri Lankan dipterocarp forest. *Journal of Vegetation Science*.

**Appendix S14.** Estimated intensity models for each life stage of each study species

A) Results for recruits. A “n” indicates a significant negative coefficient and “p” indicates a

significant positive coefficient. An empty cell means that the covariate was not included in the model.

21

**Species**

**Aspect**

**Curvature**

**Elevation**

**PC1**

**PC2**

**PC3**

**Slope**

**TWI**

ACTIAL	n***	p*

ANISCI	n***	p*** BHESCE	p**	n***	p*** CALOBR	n***	n*** CALOTH	p***	p**	n***	n***	p***	n*** CINNCA	n***	n***

CINNDU	p*	n*

CINNVE	n***	p*	n*

CRYPWI	n***	n***	p***	p***	n***	n*** CULLCE	p*	n***	n***	p*	p*	p**

CULLRO	n***	n**	p***	n***	n***	p*

DIOSAC	p***	n***	n***	n*	p***	n* DIOSHI	p**	n***	n***

DIOSQU	p**	n***	n***	p** DYSOPE	n***	n***	n***

ELAESU	n***	n***	n*	p***	n***	n***	n*** EUPHLO	n*	n***	n***	p***	p***	p** GARCHE	n**	p**	n**	n***	n***	n*** GARCMO	n**

GLENUN	p*	n***	n***	p*** GOMPSE	p***	n***	p*** GYRIWA	p***	p**	n***	n* HOPEJU	p**	p*	n***	n***	n**	p***

HUMBLA	p***	p***	p***	n***	p***	p*** HYDNOC	p*

MANGZE	p*	n***	n*	p*** MASTTE	n***	p***	n***	n*** MESU	p***	n***	n***	p***	p***	n***

MESUFE	n*	n***	n***	n***	p***	p***	p***

MYRIDA	p**	p***	n**

Note: Degree of significance: *p < 0.05, **p < 0.01, ***p < 0.001.

22

NOTHBE	p*	n***	n***	p***	p**

PALACA	n***	n**	n***	n***	n*** PALAPE	p**	n***	n***	p***	p***	p***	p* PALATH	n*	p***	p***	n***	n***	p***	p*** PODATH	n*	n***	p***	p*

PUTRRO	p**	n* RGMA	p*	p***	p**	n**	n***	n*

SEMEGA	n***	n***	n**	n***	p*	n*	n*** SEMESU	n***	n*	n***

SEMEWA	n**	n***	n***	p*	p***

SHORAF	p***	p***	p***	n***	p***	p*** SHORCN	n***	n***	p***	p***	n**	n*** SHORCR	p***	n***	p***	p*	p***	n*** SHORDI	p***	p***	n***	n**	p***	n*** SHORME	n***	n***	n***	n***	p***	p***	p***	p** SHORST	p***	p***	p*

SHORTR	n***	n***	n***	p*	p***	n***	p***	n*** SHORWO	p***	n***	n***	p*	p***	p***	n* STRO	p***

SYZYCY	n***	n*	n*

SYZYLI	n***	p**	p*	n***

SYZYMA	n***	n***

SYZYNE	n**	n***	p***	p***	n*	p**	n*** SYZYSY	n*	n***	n**

SYZYWI	n***	n**	n***	n*** TRICER	p*	n**	p***	n***	n***	n***

XYLOCH	n***	p***	n***

B) Results for juveniles. A “n” indicates a significant negative coefficient and “p” indicates a

significant positive coefficient. An empty cell means that the covariate was not included in the model.

23

**Species**

**Aspect**

**Curvature**

**Elevation**

**PC1**

**PC2**

**PC3**

**Slope**

**TWI**

ACTIAL	n***	p**	n**	n** ANISCI	p***	n***	p**	n***

BHESCE	n***	n***	n***

CALOBR	p***	n***	p***	n*** CALOTH	p**	p***	p*	n***	n***	p***	n*** CINNCA	n**	n***	p***	n*

CINNDU	n*	p*	n*	n*	n***	n*** CINNVE	n**	p**	p*** CRYPWI	n***	n***	p***	p***	p***	n***	p***	p* CULLCE	p***	p**	n***	n***	p**	p***	n*** CULLRO	p*	p***	n***	n***	p***	p***

DIOSAC	p***	n***	n***	p***	n*** DIOSHI	p**	n***	n***

DIOSQU	p*	n**	n***	p*	p*

DYSOPE	n***	n***	n***	p*** ELAESU	n***	n***	n*	p***	n*** EUPHLO	n***	n***	p***	n***

GARCHE	p***	n***	n***	p***	n*	n*** GARCMO	p*	n**	n***	n*

GLENUN	n***	n***	p***

GOMPSE	p**	n**	p***	p***	n***	p*** GYRIWA	n***	n*	p***	n***

HOPEJU	p**	n***	n*	p***	n** HUMBLA	p***	p***	p***	p***	n***	p***	p*** HYDNOC	p*	n***	p***	p*	n*** MANGZE	p***	n***	n***	p*	p***	n*** MASTTE	n***	n***	p***	n***	n*** MESU	p*	p***	n***	n***	p***	p***	n*** MESUFE	p***	n***	p***	n***	p***	p*** MYRIDA	p***	p***	p***	n***	p***	n*** NOTHBE	p***	p***	n***	p***	n***	p**

PALACA	n***	n***	n***	p***

PALAPE	n***	n***	n***	p***	p***	p*** PALATH	p***	p***	n***	p***	p***

Note: Degree of significance: *p < 0.05, **p < 0.01, ***p < 0.001.

24

PODATH	n***	p**

PUTRRO	p***	n***	n*	n*** RGMA	p***	p**	p***	n***	n***	n***	n*** SEMEGA	n***	n***	p***	n***	n*** SEMESU	n***	p**	p**	n***	n***

SEMEWA	p*	n***	p***	p*	n***	p***	p** SHORAF	p***	p***	p*	n***	p**	p***	n*** SHORCN	p**	n***	p**	p***	n**	n***

SHORCR	p***	p***	n***	n*	p*** SHORDI	p***	n***	p***	n***	n***	p***	p*** SHORME	n***	n***	n***	p***	p***	p***	p** SHORST	p***	n*	p**	p***	p***	n**	p*	n*

SHORTR	p**	n*	n***	p***	p***	n***	n**	n*** SHORWO	p***	p***	n***	p**	p***	p***	n*** STRO	p***	p**	n***	p***	p*

SYZYCY	n***	p***

SYZYLI	p*	n**	p***	p**	n**

SYZYMA	p*	n***	p*	p*** SYZYNE	p***	n***	p***	p***	n*** SYZYSY	n***	p*	n*	n***

SYZYWI	p**	n***	p***	n***	n**	n***

TRICER	n***	p***	n***	p*** XYLOCH	p**	p***	n***

C) Results for adults. A “n” indicates a significant negative coefficient and “p” indicates a significant positive coefficient. An empty cell means that the covariate was not included in the

model.

25

**Species**

**Aspect**

**Curvature**

**Elevation**

**PC1**

**PC2**

**PC3**

**Slope**

**TWI**

ACTIAL	n***	p***

ANISCI	p***	n**	n***	n***

BHESCE	p**	n**	n* CALOBR	n***

CALOTH	n***	n**	p**	n* CINNCA	n***	p***	p**

CINNDU	n*	n*	n***	n*

CINNVE	p*	n***	n* CRYPWI	n***	n***	p*	n**

CULLCE	p**	n***	n***	p***	p***	n** CULLRO	p***	n***	n***

DIOSAC	p***	p*	n***	n***	p*	p***	n** DIOSHI	p*	n***	n***	n**	n*

DIOSQU	p*	n*

DYSOPE	n***	n***	p*

ELAESU	n***	p**	p**	n***	n* EUPHLO	p*	n**	p***	n***

GARCHE	n***	p***	n***	p***	n***	n***	n*** GARCMO	p**	p***	n***	n***

GLENUN	n***	n**	p***	p* GOMPSE	p***	p***	n***	p***

GYRIWA	n***	p***	p** HOPEJU	p***	n***	n***	p*

HUMBLA	p***	p***	p***	p***	n***	p***	p***	n*** HYDNOC	n*	n***	p***	p***

MANGZE	p*	n*	p*	p** MASTTE	n***	p***	p***	n***	p***	p*

MESU	p***	p***	n***	n***	p***	p***	n*** MESUFE	p***	n***	p**	n***	n*	p***	p*** MYRIDA	p*	n***	p***	n**	n** NOTHBE	p**	n**	n***	p***	n***	p*** PALACA	n***	p*	n***	n***	p***	p***

PALAPE	p*	n***	p***	p**	p**

PALATH	p***	n***	p***	p***	n*

Note: Degree of significance: *p < 0.05, **p < 0.01, ***p < 0.001.

26

PODATH	n**	n***	n**

PUTRRO	p***	p*	n***	n* RGMA	p**	p***	n***

SEMEGA	n***	n***	n**	p***	n***	n*** SEMESU	n*	p***	n*** SEMEWA	n*	n***	p*	p***	n***

SHORAF	p***	p***	n***	p***	p***	n*

SHORCN	p***	p***	n** SHORCR	p***	p***	n**	n**	p***

SHORDI	p***	p***	n***	n**	p***	p***

SHORME	n*	n***	n***	p***	p***	p***	p***

SHORST	p***	p***	p***	n***	p***

SHORTR	p***	n***	p***	p***	n***	n* SHORWO	p***	p***	n***	p***	p***	n*** STRO	n**	n***	p***	p*** SYZYCY	n**	n***	p**	n***

SYZYLI	n*	p**	n**	n**

SYZYMA	p**	p*

SYZYNE	n*	n***	p***	p***	n***	n**

SYZYSY	p*	n***	p*	n**	n* SYZYWI	n**	n***

TRICER	n**	p***	n***	n***	n***

XYLOCH	p***	p*	p***	n**	n**	n***

**Supporting information to the paper**

Species-habitat associations in a Sri Lankan dipterocarp forest. *Journal of Vegetation Science*.

**Appendix S15.** Comparison of the strength of habitat association between life stages. The strength of habitat association was measured by the proportion of individuals located in quadrats with an intensity *λf* (*x*) < 2.5 *λf* (a – c) or < 1.5 *λf* (s – f), if the intensity would completely drove the species distribution. A low proportion means that the species is located in few very quadrats with high intensity (i.e., a generalist species), whereas high proportion means that the species is located mainly in average quality quadrats (i.e., a generalist species).

27

28

**Supporting information to the paper**

Species-habitat associations in a Sri Lankan dipterocarp forest. *Journal of Vegetation Science*.

**Appendix S16.** Examples for the normalized intensity function λf(x)/λf maps of different species across life stages at the Sinharaja plot. We normalized λf(x) with the the mean intensity λf to a mean of one across the plot. Dark blue indicate unsuitable areas and red highly suitable areas. Top: recruits, middle: juveniles, bottom: adults. The black contour line gives λf(x)/λf = 1.5, the numbers below the bottom panel give for recruits, juveniles, and adults, respectively, the proportion of individuals located in quadrats with λf(x)/λf λ(x) < 1.5 (if the intensity completely drove the species distribution), and the single number below is the correlation coefficient between the intensity functions of recruits and adults. For the species *Palaquium thwaitesii*, we show also the observed location of the trees.
